# Synergistic associations of metformin and GLP‐1 receptor agonist use with adiposity‐related cancer incidence in people living with type 2 diabetes

**DOI:** 10.1111/dom.70267

**Published:** 2025-11-03

**Authors:** Alex E. Henney, Megan Heague, David R. Riley, Theresa J. Hydes, Matthew Anson, Uazman Alam, Daniel J. Cuthbertson

**Affiliations:** ^1^ Department of Cardiovascular & Metabolic Medicine University of Liverpool Liverpool UK; ^2^ Metabolism & Nutrition Research Group Liverpool University Hospitals NHS Foundation Trust Liverpool Merseyside UK; ^3^ Liverpool Centre for Cardiovascular Sciences University of Liverpool and Liverpool University Hospitals NHS Foundation Trust Liverpool Merseyside UK; ^4^ Visiting Fellow, Centre for Biomechanics and Rehabilitation Technologies Staffordshire University Stoke‐on‐Trent UK

**Keywords:** cohort study, GLP‐1 analogue, metformin, real‐world evidence, type 2 diabetes

## Abstract

**Background:**

Metformin and glucagon‐like peptide‐1 receptor agonists (GLP‐1 RAs) may reduce the risk of adiposity‐related cancers in patients with type 2 diabetes (T2D). The potential synergistic effects of these treatments on cancer incidence remain unclear, considering their distinct biological mechanisms and their associated effects on body weight.

**Methods:**

A retrospective cohort analysis was conducted using a large global database (TriNetX) of patients with T2D. Three cohorts: single agent use with metformin or GLP‐1 RAs, and dual/combination metformin/GLP‐1 RA use, were compared with a reference group treated with DPP4 inhibitors (DPP4i). Propensity score matching (1:1) was applied to control for confounders. Cancer incidence and all cause mortality were assessed over 5 years of follow up.

**Results:**

After matching, metformin and GLP‐1 RA treatment were both associated with a lower risk of all adiposity‐related cancers and all‐cause mortality; the latter was associated with greater reductions in both. Cancer rates were (hazard ratio) 0.96 [95% CI 0.92, 0.99] and 0.86 [0.82, 0.89], while mortality rates were 0.78 [0.76, 0.80] and 0.61 [0.59, 0.63] for metformin and GLP‐1 RA, respectively. Dual therapy showed the strongest association with lower cancer incidence (0.61 [0.57, 0.65]) and mortality (0.33 [0.32, 0.35]). Results were more significant in younger, male patients with obesity.

**Conclusion:**

In patients with T2D, dual metformin and GLP‐1 RA treatment was associated with a 39% lower incidence of adiposity‐related cancers and a 67% lower mortality, with a striking impact on cancer‐related outcomes.

## 1 INTRODUCTION

Obesity is a major risk factor for cardiovascular disease, metabolic dysfunction‐associated steatotic liver disease (MASLD) and type 2 diabetes (T2D), but also an independent risk factor for cancer.^1^ Between 4% and 8% of cancers are attributed to living with overweight/obesity (adiposity‐related cancers), the leading cancer risk factor after smoking.^2^ At least 13 types of adiposity‐related cancer are reproducibly reported, including endometrial, colorectal, breast cancer, oesophageal, renal, pancreatic, liver, myeloma, ovarian and thyroid, with the risk much higher in women (endometrial, postmenopausal breast, and colorectal cancers account for >60% of cases).^3^ Recently, unpublished data suggest other site‐specific cancers are adiposity‐related.^4^ Importantly, meta‐analysis has highlighted an incremental association between weight gain and risk of endometrial, ovarian, breast, colorectal, and renal cancer.^5^ The obesity‐associated risk is influenced more by waist circumference than BMI, hence the term adiposity‐related cancer.^6^

The stark increase in obesity constitutes a major determinant of the increasing cancer rates, particularly in the young.^7^ Data from Cancer Research UK shows a doubling of the rates of obesity in the last three decades coinciding with increased cancer incidence, with a 19% and 22% increase in <25‐ and 25–49‐year‐old people, respectively, with further projected increases for most types of cancer. Of the five cancers with the highest incidence rates, three of these are amongst the 13 adiposity‐related cancers, in both men and women. The increased adiposity‐related cancer risk is further amplified by the presence of T2D, underlying why cancer and other non‐vascular diseases, have become a major cause of morbidity and mortality in these patients.^8^, ^9^ This is particularly important considering the rise in young‐onset type 2 diabetes.^10^

Furthermore, weight loss may also reduce cancer risk, following both surgical and non‐surgical approaches, particularly for female adiposity‐related cancers.^11^ Current evidence suggests metabolic surgery is most efficacious, associated with a reduced overall incidence of cancer (by 38%), adiposity‐related cancer (by 41%) and cancer‐associated mortality (by 49%).^12^ Given the strong association between obesity and cancer, it is plausible that weight loss may be a viable intervention for reducing cancer incidence. A reduction in cancer risk has also been observed for glucose‐ and weight‐lowering medications. Metformin, a first‐line treatment for T2D, has been shown in multiple meta‐analyses of cohort studies, to decrease cancer incidence by 30%–40%; however, excluding time‐biased studies, the reduction in cancer incidence is more modest.^13^ Hence, meta‐analysis of clinical trials has failed to reproduce such results.^14^ More recently, there is emerging evidence suggesting GLP‐1 receptor agonists (GLP‐1 RAs) are associated with a lower incidence of cancer in patients treated for obesity and/or T2D.^15^ This benefit is countered by concerns regarding the possible association between GLP‐1 RAs and pancreatic/thyroid cancer.^16^, ^17^

Given conflicting evidence for the effects of metformin and GLP‐1 receptor agonists, we utilized real‐world data to determine the individual and synergistic effects of both metformin and GLP‐1 RAs on the risk of adiposity‐related cancers in patients with T2D.

## 2 METHODS

### 2.1 Study design

We conducted a cohort study with anonymised data from TriNetX (TriNetX LLC, Cambridge, MA, USA), a global federated health research network with access to both inpatient and outpatient electronic medical records from health care organisations internationally, primarily secondary, and tertiary care providers in North America and Western Europe. This analysis was conducted on the Global Collaborative Network, containing data from >135 million patients with access to diagnoses, procedures, medications, laboratory values and genomic information worldwide. Data were collected on 7th June 2024. Further details on the network are described elsewhere.^18^

### 2.2 Population

**FIGURE 1:**
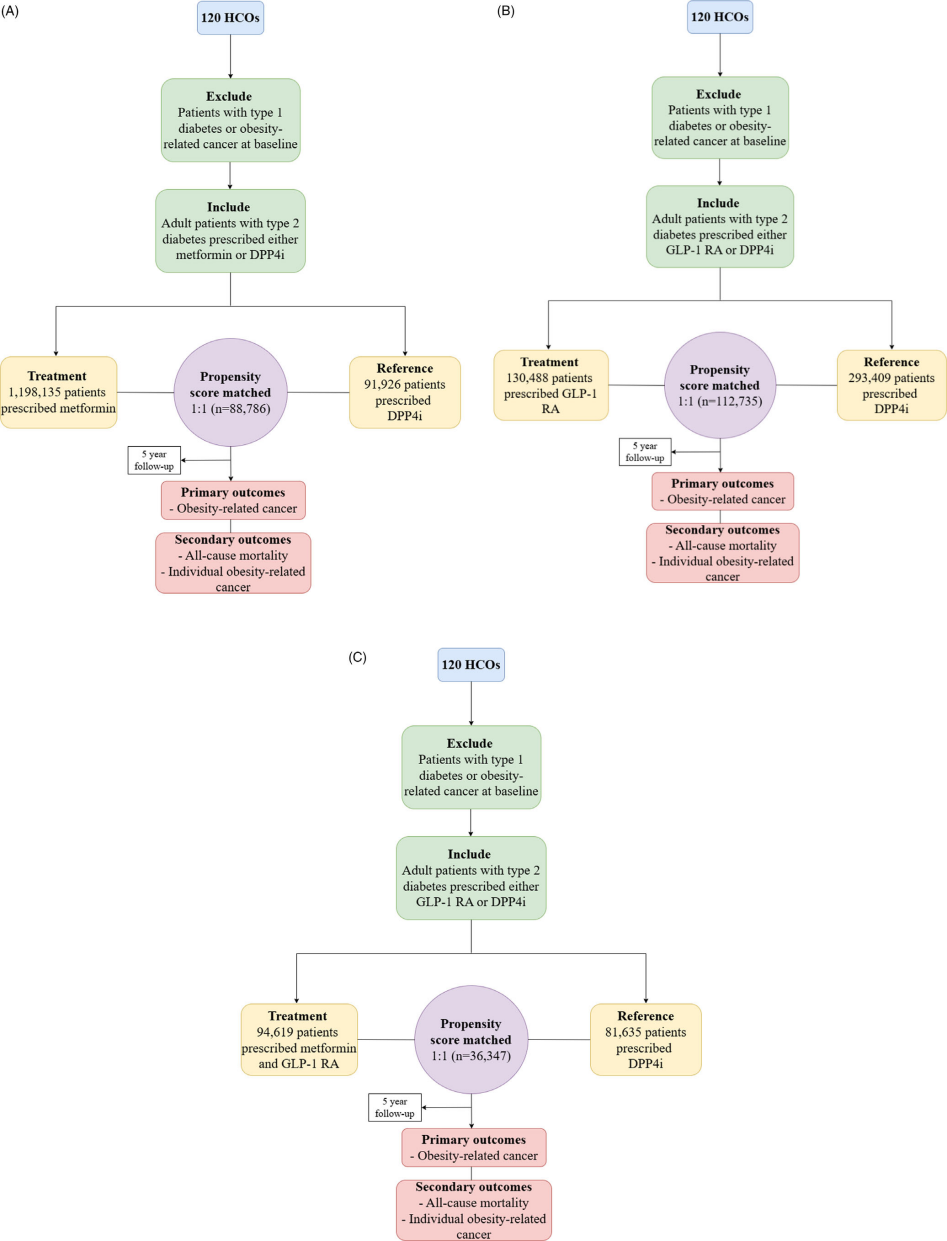
Study flow diagram for (A) patients prescribed metformin vs. DPP4i, (B) patients prescribed GLP‐1 RA vs. DPP4i, and (C) patients prescribed dual metformin + GLP‐1 RA treatment vs. DPP4i.

We identified adults (>18 years) with type 2 diabetes (ICD‐10 code E11) in TriNetX. Two arms were generated for each cohort: a treatment and a reference arm. Within the treatment arm, three cohorts were created: (1) Cohort 1, metformin, (2) Cohort 2, GLP‐1 RA, and (3) Cohort 3, dual therapy with both metformin and GLP‐1 RA. Metformin and GLP‐1 RAs were chosen as the treatment agents given their pleiotropic clinical effects, beyond glucose lowering. DPP4 inhibitors were chosen as the reference treatment arm considering their neutral effect on body weight and cancer incidence.^19^ Patients were excluded if they had a history of type 1 diabetes (ICD‐10 code E10), or if, prior to initiation of the respective treatment, they had a diagnosis of adiposity‐related cancer (hepatocellular, colorectal, gastric, pancreatic, oesophageal, gallbladder, kidney, thyroid, brain, myeloma, breast, uterine, ovarian, oral cavity, nasal and sinus, adrenal, parathyroid, pituitary, connective tissue, head and neck, penis, melanoma, vulva, cervical). The treatment arm additionally excluded patients who had previously been prescribed the reference arm treatment and vice versa (Figure 1). Definitions of all diagnoses are presented in Table S1.

### 2.3 Index event

The index event followed an active comparator new user design where the analysis was of new starters of each drug. This served to reduce the risk of time‐related bias present in previous observational studies assessing metformin's association with cancer incidence.^20^ Patients were followed up for 5 years from the first day of starting the new drug (treatment or reference). All analyses were conducted using an intention‐to‐treat approach from the date of the index prescription.

### 2.4 Propensity score matching

Cohorts were propensity score matched (PSM), in a 1:1 ratio, for age, sex, ethnicity, smoking, socioeconomic status, cardiovascular disease (ischaemic heart disease (IHD), cerebrovascular accident (CVA) and peripheral vascular disease (PVD)), body mass index (BMI), estimated glomerular filtration rate (eGFR), glycated haemoglobin (HbA1c), presence of hypertension, dyslipidaemia, or previous non‐obesity‐related cancer, and other blood glucose lowering therapies. Definitions for PSM covariates are presented in Table S1. Anthropometric values, including BMI, were obtained from vital sign measurements recorded in the EHR from associated HCO.

### 2.5 Outcomes

The *primary outcome* of interest was *time‐to‐incident composite adiposity‐related cancer*. The composite outcome was grouped as all adiposity‐related cancers (hepatocellular, colorectal, gastric, pancreatic, oesophageal, gallbladder, kidney, thyroid, brain, myeloma, breast, uterine, ovarian, oral cavity, nasal and sinus, adrenal, parathyroid, pituitary, connective tissue, head and neck, penis, melanoma, vulva, cervical), and traditional adiposity‐related cancers (hepatocellular, colorectal, gastric, pancreatic, oesophageal, gallbladder, kidney, thyroid, brain, myeloma, breast, uterine, ovarian).

The *secondary outcomes* of interest included *time‐to‐incidence* of the *individual adiposity‐related cancers*, as well as *all‐cause mortality*, a surrogate of overall survival (OS). Definitions used in the identification of outcomes are presented in Table S1. Both arms were followed up until the first coding of the outcome of interest on their electronic medical records. Patients who did not develop incident cancer were censored at: (i) date of death; (ii) end of data collection; (iii) loss to follow‐up.

### 2.6 Statistical analysis

Statistical analysis for cohort data was performed in situ within the TriNetX platform. PSM was performed using logistic regression. TriNetX uses ‘greedy nearest‐neighbour matching’ with a calliper of 0.1 pooled standard deviations and difference between propensity scores <0.1. We assessed covariate balance between groups using the standardised mean difference (SMD). SMD <0.1 was considered well matched. Survival analysis was performed to estimate the probability of an outcome, at daily time intervals, over 5 years from the index event. A hazard ratio (HR), log rank test and Kaplan–Meier survival curve were generated. TriNetX uses the R Survival package v3.2‐3.

### 2.7 Sensitivity analyses

Additionally, for sensitivity analysis, we performed a stratified analysis by body mass index (presence or absence of obesity: ICD‐10 coding (E66), or BMI >30 vs. <30 kg/m^2^), age (older adults vs. younger adults: >60 vs. < 60 years), ethnicity (white vs. non‐white ethnic background), sex (male vs. female), specific drug (semaglutide vs. liraglutide), type 2 diabetes duration (≥/<10 years), presence of alcohol‐use disorders (ICD‐10 code F10), diagnosis of adiposity‐related cancers within the first 6 months following index were excluded (to ensure exclusion of patients with cancer development prior to the initiation of treatment), and glucose treatment adherence (persistence of medication use) data is not directly available within TriNetX so it is not possible to determine individuals' duration of treatment. To account for the important matter of treatment adherence, we mandated at least one repeat prescription code, at least 1 year after the index date, to enrich for persistent treatment exposure. Additionally, we calculated E‐values, representing the minimum strength of association on the HR scale that an unmeasured confounder would need to have with both the exposure (treatment arm) and the outcome, conditional on the measured confounders, to explain away the observed association; HR + √[HR×(HR‐1)].^21^ The Strengthening the Reporting of Observational Studies in Epidemiology guidelines were followed in the reporting of this cohort study.^22^

## 3 RESULTS

### 3.1 Cohort 1, Metformin

1 290 061 patients were identified: 1198135 (92.9%) prescribed metformin, and 91 926 (7.1%) prescribed DPP4is. The metformin arm was younger, with a higher eGFR and BMI, and less likely to have comorbidities: hypertension, IHD, CVA and PVD (Table 1). After PSM, each cohort was deemed well matched. The total number of participants in each cohort was reduced to 88 786 (Table 1).

**TABLE 1:** Baseline characteristics for all three cohorts.

	Before propensity score matching			After propensity score matching		
Characteristic	Treatment	Reference	SMD	Treatment	Reference	SMD
*Metformin vs. DPP4i*						
Demographics						
Numbers (n)	1 198 135	91 926		88 786	88 786	
Age (years)	58.8 ± 14.1	67.9 ± 12.9	0.67	67.9 ± 12.6	67.9 ± 12.9	0.02
Sex, female (%)	48.6	48.6	<0.01	49.9	48.5	0.03
Ethnicity, white (%)	60.6	57.9	0.06	59.6	58.4	0.02
Adverse socioeconomic markers (%)	1.3	0.8	0.05	0.8	0.8	<0.01
Anthropometrics						
Body mass index (kg/m^2^)	33.5 ± 8.2	31.2 ± 7.6	0.30	31.3 ± 7.7	31.3 ± 7.6	<0.01
Biochemistry						
HbA1c (%)	7.6 ± 2.1	7.6 ± 1.9	0.02	7.7 ± 2.1	7.6 ± 2.0	0.05
Glomerular filtration rate (ml/min/1.73 m^2^)						
>90			0.35			0.01
80–90			0.21			<0.01
70–80			0.12			<0.01
60–70			0.04			0.01
50–60			0.28			0.01
40–50			0.47			0.01
30–40			0.54			<0.01
20–30			0.49			0.02
15–20			0.36			0.03
<15			0.27			0.02
Comorbidity (%)						
Non‐obesity‐related cancer	14.4	14.5	<0.01	14.0	14.2	0.01
Ischaemic heart disease	14.7	27.3	0.31	26.3	26.3	<0.01
Cerebrovascular accident	6.5	11.8	0.18	11.5	11.3	0.01
Peripheral vascular disease	2.5	5.8	0.17	5.3	5.4	<0.01
Hypertension	53.7	62.4	0.18	60.8	61.5	0.01
Dyslipidaemia	42.4	46.8	0.09	44.9	46.1	0.02
Medication						
Insulin	26.5	44.9	0.39	43.3	43.4	<0.01
Sulphonylureas						
Glipizide	9.3	14.7	0.17	14.3	14.3	<0.01
Glimepiride	5.1	10.9	0.21	10.7	10.6	0.01
Glyburide	4.2	3.3	0.05	3.2	3.3	<0.01
PPAR gamma agonists						
Pioglitazone	3.2	6.4	0.15	6.2	6.2	<0.01
Rosiglitazone	0.5	0.5	<0.01	0.6	0.5	0.01
SGLT2 inhibitors						
Empagliflozin	0.8	1.5	0.06	1.7	1.5	0.02
Dapagliflozin	0.6	0.6	<0.01	0.7	0.6	0.01
Canagliflozin	0.9	1.2	0.03	1.3	1.3	0.01
GLP1 receptor agonists						
Liraglutide	1.6	1.1	0.04	1.2	1.1	<0.01
Semaglutide	0.3	1.5	0.03	0.3	0.2	0.02
Exenatide	0.8	0.7	0.01	0.7	0.7	<0.01
Dulaglutide	0.8	0.6	0.03	0.7	0.6	0.01
Acarbose	0.2	0.7	0.08	0.3	0.6	0.05
*GLP‐1 RA vs. DPP4i*						
Demographics						
Numbers (n)	130 488	293 409		112 735	112 735	
Age (years)	55.9 ± 12.0	64.4 ± 12.9	0.68	57.5 ± 11.4	57.4 ± 12.3	0.01
Sex, female (%)	53.4	46.0	0.15	51.6	51.3	0.01
Ethnicity, white (%)	67.1	56.5	0.22	65.5	66.9	0.03
Adverse socioeconomic markers (%)	1.5	1.0	0.05	1.3	1.3	0.01
Anthropometrics						
Body mass index (kg/m^2^)	37.4 ± 8.1	31.5 ± 7.4	0.76	36.6 ± 8.0	35.5 ± 7.7	0.14
<20			0.09			0.01
20–25			0.32			0.02
25–30			0.25			0.01
30–35			0.04			0.01
35–40			0.25			<0.01
>40			0.36			0.02
Biochemistry						
HbA1c (%)	8.3 ± 2.1	8.0 ± 2.0	0.14	8.2 ± 2.1	8.3 ± 2.1	0.05
Glomerular filtration rate (ml/min/1.73 m^2^)						
>90			0.16			0.02
80–90			0.09			0.01
70–80			0.05			0.01
60–70			0.04			0.01
50–60			0.12			0.01
40–50			0.17			0.01
30–40			0.18			0.01
20–30			0.17			0.01
15–20			0.13			<0.01
<15			0.06			<0.01
Comorbidity (%)						
Non‐obesity‐related cancer	16.9	15.8	0.03	16.4	15.7	0.02
Ischaemic heart disease	14.9	21.9	0.18	15.9	15.5	0.01
Cerebrovascular accident	5.4	10.0	0.17	5.9	5.6	0.01
Peripheral vascular disease	3.1	4.2	0.06	3.2	3.2	<0.01
Hypertension	59.2	60.9	0.03	59.3	57.9	0.03
Dyslipidaemia	55.1	51.0	0.08	53.9	52.5	0.03
Medication						
Metformin	63.3	61.6	0.04	62.8	62.0	0.02
Insulin	45.0	34.6	0.21	42.0	41.6	0.01
Sulphonylureas						
Glipizide	14.3	16.7	0.07	14.7	14.8	0.01
Glimepiride	10.7	12.8	0.07	11.0	10.9	<0.01
Glyburide	5.0	6.2	0.05	5.2	5.0	0.01
PPAR gamma agonists						
Pioglitazone	7.8	8.3	0.02	7.9	8.0	<0.01
Rosiglitazone	0.8	1.1	0.03	0.8	1.0	0.02
SGLT2 inhibitors						
Empagliflozin	5.1	1.6	0.19	3.7	3.4	0.02
Dapagliflozin	3.3	0.9	1.67	2.2	2.0	0.01
Canagliflozin	5.4	1.7	0.20	3.9	3.5	0.02
Acarbose	0.4	0.9	0.07	0.4	0.6	0.03
*Dual metformin and GLP‐1 RA treatment vs. DPP4i*						
Demographics						
Numbers (n)	94 619	81 635		36 347	36 347	
Age (years)	53.8 ± 12.0	68.8 ± 12.7	1.21	60.8 ± 10.3	60.9 ± 11.6	0.01
Sex, female (%)	52.6	47.8	0.09	49.1	48.8	<0.01
Ethnicity, white (%)	67.5	57.1	0.22	63.6	63.9	0.01
Adverse socioeconomic markers (%)	0.9	0.8	0.02	0.7	0.7	<0.01
Anthropometrics						
Body mass index (kg/m^2^)	37.3 ± 8.1	30.7 ± 7.5	0.85	34.8 ± 7.7	34.1 ± 7.7	0.09
Biochemistry						
HbA1c (%)	8.2 ± 2.2	7.5 ± 1.9	0.33	8.0 ± 2.2	7.9 ± 2.1	0.05
Glomerular filtration rate (ml/min/1.73 m^2^)						
>90			0.36			<0.01
80–90			0.18			0.01
70–80			0.07			0.02
60–70			0.12			0.01
50–60			0.37			0.01
40–50			0.54			<0.01
30–40			0.60			<0.01
20–30			0.53			0.01
15–20			0.38			0.01
<15			0.30			0.01
Comorbidity (%)						
Non‐obesity‐related cancer	12.4	14.5	0.06	12.1	12.4	0.01
Ischaemic heart disease	10.6	28.4	0.46	17.0	17.0	<0.01
Cerebrovascular accident	3.6	12.4	0.33	6.1	6.1	<0.01
Peripheral vascular disease	1.8	6.0	0.22	3.0	3.0	<0.01
Hypertension	50.5	63.0	0.25	53.4	53.8	0.01
Dyslipidaemia	45.5	46.4	0.02	42.6	43.3	0.01
Medication						
Insulin	34.4	45.3	0.22	37.3	37.6	0.01
Sulphonylureas						
Glipizide	11.1	14.4	0.10	12.9	12.9	<0.01
Glimepiride	8.6	10.5	0.06	9.8	9.8	<0.01
Glyburide	4.7	3.2	0.08	3.6	3.8	0.01
PPAR gamma agonists						
Pioglitazone	6.3	6.1	0.01	6.4	6.6	0.01
Rosiglitazone	0.6	0.5	0.01	0.6	0.5	0.01
SGLT2 inhibitors						
Empagliflozin	3.9	1.1	0.18	2.3	2.1	0.01
Dapagliflozin	2.9	0.5	0.19	1.1	0.9	0.01
Canagliflozin	4.2	0.9	0.01	1.9	1.8	0.01
Acarbose	0.2	0.7	0.07	0.3	0.5	0.04

#### 3.1.1 Incidence of adiposity‐related cancer and mortality

**FIGURE 2:**
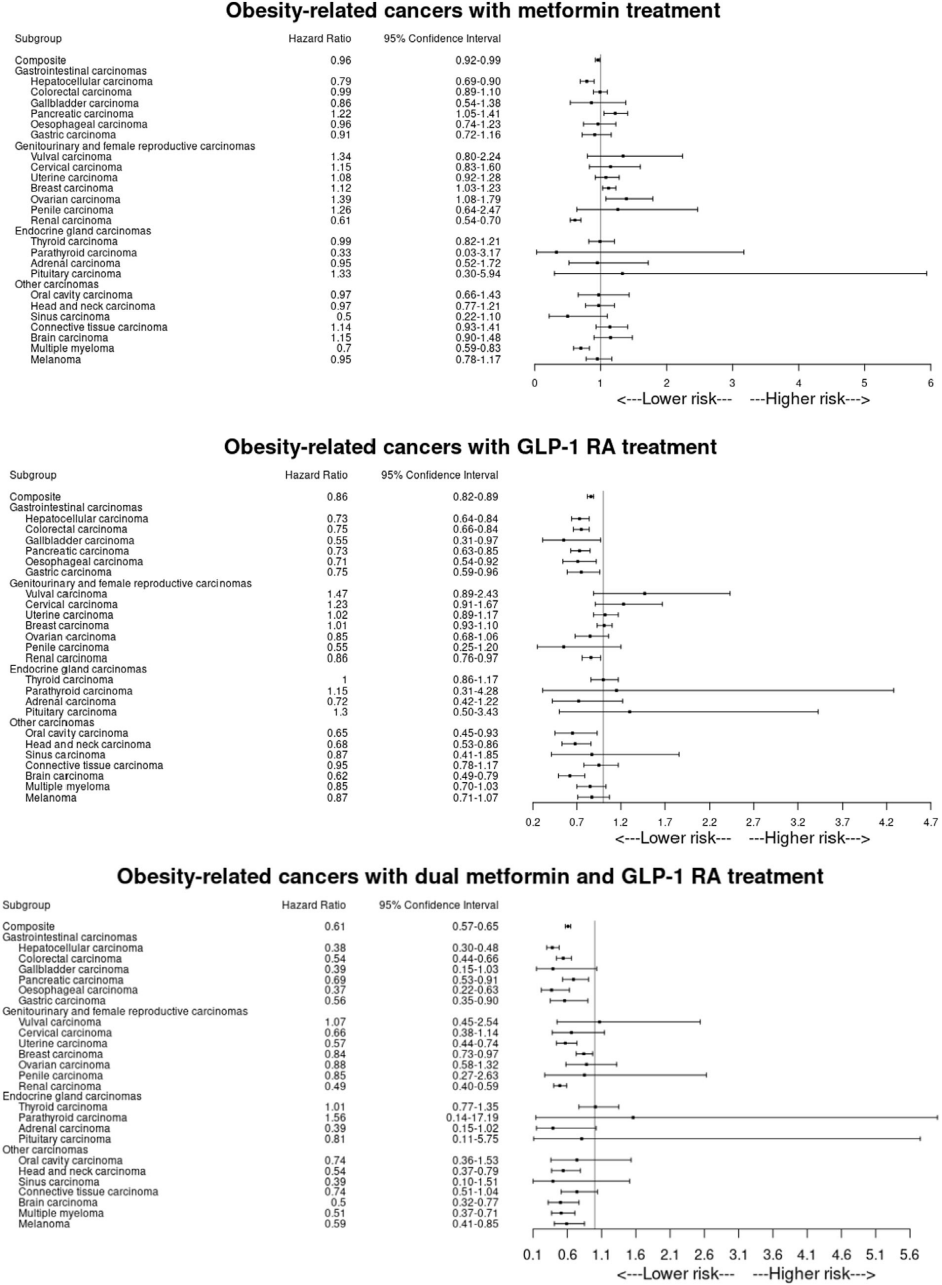
Forest plot demonstrating hazard of incident obesity‐related cancer, by stratified analysis. (A) Metformin, (B) GLP1‐RA, and (C) dual treatment with metformin and GLP‐1 RA.

**FIGURE 3:**
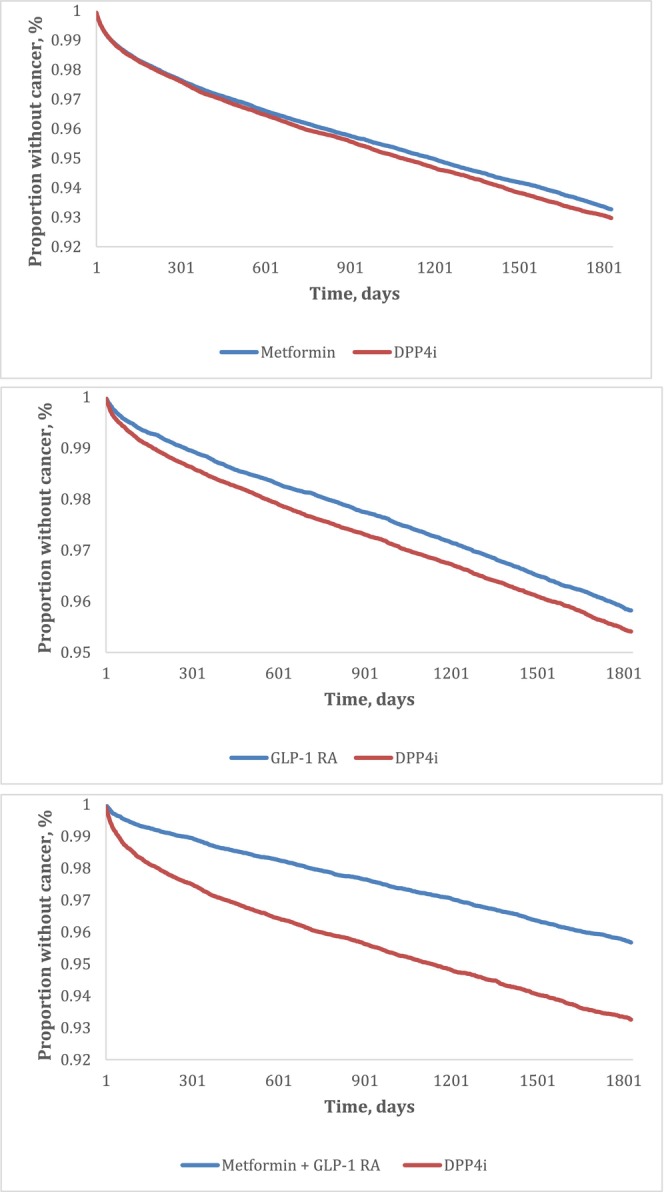
Survival curves demonstrating proportion of people without obesity‐related cancer after 5 years with (A) Metformin, (B) GLP1‐RA and (C) metformin and GLP1‐RA combination treatment, compared with DPP4i.

Treatment with metformin reduced the risk of incident composite adiposity‐related cancers; both for all adiposity‐related cancers (HR 0.96 [95% CI 0.92, 0.99]), and for traditional adiposity‐related cancers (0.95 [0.91, 0.99]) (Table 2). A forest plot is presented in Figure 2, whilst survival curves are presented in Figure 3. The incidence rate in the metformin arm for all adiposity‐related cancers was 10.0 per 1000 person‐years (vs. 10.3 in the DPP4i arm), and 8.7 (vs. 9.1 in the DPP4i arm) for traditional obesity‐related cancers. Specifically, treatment with metformin significantly reduced the risk of hepatocellular (0.79 [0.69, 0.90]), and kidney (0.61 [0.54, 0.70]), cancer, as well as multiple myeloma (0.70 [0.59, 0.83]). However, metformin was associated with increased risk of pancreatic (1.22 [1.05, 1.41]), breast (1.12 [1.03, 1.23]), and ovarian (1.39 [1.08, 1.79]), cancer. Incident mortality was also significantly lower in the metformin arm (0.78 [0.76, 0.80]). All secondary outcomes are presented in Table S2, with a forest plot presented in Figure 2.

**TABLE 2:** Outcomes of the traditional and all obesity‐related cancers with metformin, GLP1‐RA or dual treatment compared with DPP4 inhibitor.

	Sample size	Outcome (*n*)	5‐year survival probability (%)	Hazard ratio (95% confidence interval)	Log‐Rank test	*p* value	*E* value
Metformin vs. DPP4i							
*All obesity‐related cancers*							
Reference	88 786	4583	93.0	1.00 (1.00–1.00)			
Metformin	88 786	4423	93.3	*0.96* (*0.92, 0.99*)	4.7	0.03	1.25
*Traditional obesity‐related cancers*							
Reference	88 786	4035	93.8	1.00 (1.00–1.00)			
Metformin	88 786	3862	94.1	*0.95* (*0.91, 0.99*)	5.8	0.02	1.29
GLP‐1 RA vs. DPP4i							
*All obesity‐related cancers*							
Reference	112 735	4875	94.6	1.00 (1.00–1.00)			
GLP‐1 RA	112 735	4515	95.2	*0.86* (*0.82, 0.89*)	57.2	<0.01	1.60
*Traditional obesity‐related cancers*							
Reference	112 735	4302	95.2	1.00 (1.00–1.00)			
GLP‐1 RA	112 735	3979	95.8	*0.86* (*0.82, 0.89*)	51.1	<0.01	1.60
Dual metformin plus GLP‐1 RA treatment vs. DPP4i							
*All obesity‐related cancers*							
Reference	36 347	1831	93.3	1.00 (1.00–1.00)			
Metformin plus GLP‐1 RA	36 347	1356	95.7	*0.61* (*0.57, 0.65*)	196.0	<0.01	2.66
*Traditional obesity‐related cancers*							
Reference	36 347	1614	94.0	1.00 (1.00–1.00)			
Metformin plus GLP‐1 RA	36 347	1190	96.2	*0.61* (*0.56, 0.65*)	175.5	<0.01	2.66

#### 3.1.2 Stratified analysis

Results are presented in Table S5, and as a forest plot in Figures S1–S6. Treatment with metformin reduced the risk of incident all adiposity‐related cancers in (i) younger adults more than that in older adults (HR 0.71 [0.59, 0.85] vs. HR 0.96 [0.92, 0.99]); (ii) males (HR 0.93 [0.87, 0.99]), but not females (HR 1.00 [0.94, 1.06]); (iii) patients without alcohol misuse‐related disorder (HR 0.96 [0.92, 0.99]); and (iv) in patients with a shorter duration of T2D (<10 years T2D, HR 0.94 [0.90, 0.98]), but not in patients with >10 years T2,D (HR 0.95 [0.88, 1.01]).

### 3.2 Cohort 2, GLP‐1 RA

423897 patients were identified: 130488 (30.8%) prescribed GLP‐1 RAs, and 293 409 (69.2%) prescribed DPP4is. The GLP‐1 RA treatment arm patients were younger, more likely to be of white ethnicity, female and with a higher BMI, HbA1c, and eGFR, as well as less cardiovascular disease, compared with the DPP4i arm (Table 1). After PSM, each cohort was deemed well matched. The total number of participants in each cohort was reduced to 112 735 (Table 1).

#### 3.2.1 Incidence of adiposity‐related cancer and mortality

Treatment with GLP‐1 RA reduced the risk of incident composite adiposity‐related cancers; both for all adiposity‐related cancers (HR 0.86 [95% CI 0.82, 0.89]), and for traditional adiposity‐related cancers (0.86 [0.82, 0.89]) (Table 2). A forest plot is presented in Figure 2, and survival curves in Figure 3. The incidence rate in the GLP‐1 RA arm for all adiposity‐related cancers was 8.0 per 1000 person‐years (vs. 8.7 in the DPP4i arm), and 7.1 (vs. 7.6 in the DPP4i arm) for traditional adiposity‐related cancers. Specifically, treatment with GLP‐1 RAs reduced the risk of hepatocellular (0.73 [0.64, 0.84]), colorectal (0.75 [0.66, 0.84]), gallbladder (0.55 [0.31, 0.97]), pancreatic (0.73 [0.63, 0.85]), oesophageal (0.71 [0.54, 0.92]), stomach (0.75 [0.59, 0.96]), kidney (0.86 [0.76, 0.97]), brain (0.62 [0.49, 0.79]), oral cavity (0.65 [0.45, 0.93]), and head and neck (0.68 [0.53, 0.86]) cancers. Incident mortality was also significantly lower in the GLP‐1 RA arm (0.61 [0.59, 0.63]). All secondary outcome analyses are presented in Table S3, with a forest plot presented in Figure 2.

#### 3.2.2 Stratified analysis

Results are presented in Table S5, and as a forest plot in Figures S1–S6. Treatment with GLP‐1 significantly reduced the risk of incident all adiposity‐related cancers in: (i) those with obesity (HR 0.87 [0.81, 0.92]), and healthy weight (HR 0.86 [0.75, 0.98]); (ii) younger (0.77 [0.69, 0.86]), and, to a lesser extent, older (HR 0.85 [0.81, 0.89]) adults; (iii) white (HR 0.83 [0.80, 0.88]), and not non‐white (HR 0.87 [0.81, 0.94]), ethnic backgrounds; (iv) men (0.71 [0.67, 0.76]), and, to a lesser extent, women (0.88 [0.84, 0.93]); (v) semaglutide (0.78 [0.65, 0.94]), and, to a lesser extent, liraglutide (0.88 [0.83, 0.94]); (vi) patients without a history of excess alcohol misuse (0.85 [0.81, 0.89]); and (vii) patients with a diagnosis of diabetes for >10 years (0.79 [0.74, 0.85]), and < 10 years (0.84 [0.80, 0.88]).

### 3.3 Cohort 3, Dual metformin and GLP‐1 RA

176254 patients were identified: 94619 (53.7%) prescribed dual metformin and GLP‐1 RA treatment, and 81 635 (46.3%) prescribed DPP4is. The dual metformin and GLP‐1 RA treatment arm was younger, more likely to be of white ethnicity, and have a higher BMI, HbA1c, and eGFR, but less likely to have CVD, hypertension, or dyslipidaemia, compared with the DPP4i arm (Table 1). After PSM, cohorts were well matched. The total number of participants in each cohort was reduced to 36 347 (Table 1).

#### 3.3.1 Incidence of adiposity‐related cancer and mortality

Dual treatment reduced the risk of incident composite adiposity‐related cancers; both for all adiposity‐related cancers (HR 0.61 [95% CI 0.57, 0.65]), and for traditional adiposity‐related cancers (0.61 [0.56, 0.65]) (Table 2). A forest plot is presented in Figure 2, and survival curves in Figure 3. The incidence rate in the dual metformin and GLP‐1 RA treatment arm for all obesity‐related cancers was 7.5 per 1000 person‐years (vs. 10.1 in the DPP4i arm), and 6.6 (vs. 8.9 in the DPP4i arm) for traditional obesity‐related cancers. Specifically, dual treatment with metformin and GLP‐1 RAs reduced the risk of hepatocellular (0.38 [0.30, 0.48]), colorectal (0.54 [0.44, 0.66]), pancreatic (0.69 [0.53, 0.91]), oesophageal (HR 0.37 [95% CI 0.22, 0.63]), stomach (0.56 [0.35, 0.90]), uterine (0.57 [0.44, 0.74]), breast (0.84 [0.73, 0.97]), kidney (0.49 [0.40, 0.59]), brain (0.50 [0.32, 0.7]), multiple myeloma (0.51 [0.37, 0.71]), head and neck (0.54 [0.37, 0.79]) cancers, and melanoma (0.59 [95% CI 0.41, 0.85]). Incident mortality was also lower in the dual metformin and GLP‐1 RA treatment arm (HR 0.33 [95% CI 0.32, 0.35]). All outcome analysis results are presented in Table S4, with a forest plot presented in Figure 2.

#### 3.3.2 Stratified analysis

Results are presented in Table S3, and as a forest plot in Figures S1–S6. Dual treatment with metformin and GLP‐1 was associated with a significantly lower incidence of all obesity‐related cancers, in all stratified analyses.

## 4 DISCUSSION

In this large real‐world cohort, we demonstrate that in patients with T2D, metformin treatment was associated with a modest (4%) reduction in the risk of all adiposity‐related cancers (specifically, a lower risk of hepatocellular, kidney, and myeloma) with more robust risk reduction in younger adults living with obesity. However, metformin use was associated with an increased risk of thyroid, breast, and ovarian cancer. GLP‐1 RA use was associated with a greater reduction in risk (14%), driven specifically by a lower risk of hepatocellular, colorectal, gallbladder, gastric, pancreas, oesophageal, brain, oral cavity, and head and neck cancers. Semaglutide offered the most pronounced chemoprotective effects, with the effects of all GLP‐1 RAs generally greater in younger adult patients of white ethnicity, living with obesity, and in those with longer duration T2D. Crucially, a combination of metformin and GLP‐1 dual treatment had a staggering 39% lower risk of all‐adiposity related cancers.

Meta‐analyses of observational studies suggest that metformin reduces cancer incidence in patients with T2D, with common characteristics shared between these and our findings. Risk reductions were seen in similar site‐specific cancers (hepatocellular and renal cell carcinoma, and myeloma),^23^, ^24^, ^25^ and the cancer subtypes where metformin appears chemoprotective, were independently associated with T2D,^26^ suggesting glycaemic control may be implicated in their pathophysiology. However, given the use of DPP4i as the reference arm, and robust propensity matching for HbA1c, it seems unlikely that improved glycaemic control is the main mechanism underpinning the chemoprotection observed presently. The validity of previous cohort studies included in meta‐analyses suggesting that metformin use is associated with a reduced cancer risk has been questioned.^27^, ^28^, ^29^ There are concerns regarding poor methodological design resulting in time‐related bias that may exaggerate the magnitude of the effect size reported. Studies that have deployed similar active comparator new user designs to our study, to reduce time‐related bias, are generally consistent with the analyses presented here in that metformin is associated with modest chemoprotection at best.^30^ Furthermore, in RCTs investigating the use of metformin for cancer prevention, chemoprotective effects have not been demonstrated.^14^, ^31^ Finally, the apparent higher incidence of breast and ovarian cancer with metformin should be interpreted with caution. Although these findings may be real, they may reflect residual confounding by factors such as screening practices, which are not captured within TriNetX. Given the number of site‐specific analyses, chance findings cannot be excluded. Notably, in the dual‐therapy group (with combination GLP‐1 RA and metformin therapy), breast cancer risk was lower, an observation that would not be consistent with a causal adverse effect of metformin.

Evidence surrounding GLP‐1 RAs and associated cancer risk is more scarce given their novelty; however, there is evidence of reduced risk of colorectal and hepatocellular carcinoma.^15^, ^32^ Additionally, unpublished data from 34 000 participants found that patients treated with GLP‐1 RAs were 19% less likely to develop 13 types of adiposity‐related cancers. Despite this, there remain concerns regarding possible associations between GLP‐1 RAs and increased risk of breast,^33^ pancreatic,^29^ and thyroid cancer.^16^, ^34^, ^35^ The concern for pancreatic cancer arises from increased risk of pancreatitis in observational studies and increased beta cell mass in animal models.^17^, ^36^ For thyroid cancer, GLP‐1 RAs have been found to stimulate thyroid C‐cell proliferation in rodents and GLP‐1 receptors have been identified in human neoplastic thyroid C cells and papillary thyroid carcinoma.^37^, ^38^ Meta‐analyses of clinical trials refute these mechanistic claims in human data, suggesting that GLP‐1 RAs do not increase the risk of thyroid, pancreatic, breast, or any other cancer.^39^, ^40^, ^41^ Our results are consistent with these reassuring findings.

Crucially, for the first time, we highlight a novel synergistic association of dual metformin and GLP‐1 RA treatment with (reduced) cancer incidence in patients with T2D, both with and without obesity. These associations should be interpreted as hypothesis‐generating, and, considering the observational nature of our study, causality cannot be inferred. Consistent associations of our effects across several cancer sites are likely to be both mediated by weight‐loss dependent and weight‐loss independent effects through a variety of biological mechanisms. The greater reductions in cancer rates with GLP‐1 RA therapy perhaps lend support to weight loss being an important driver of this associated protection. Despite this, the exact underlying mechanism behind the synergistic effect is unknown but may be explained by distinct physiological mechanisms of both drugs, addressing multiple oncogenic pathways associated with T2D and adiposity‐related cancers. For metformin, several putative mechanisms underlie its chemoprotection. Indirect pathways include weight maintenance which may attenuate the risk of carcinogenesis. Additionally, metformin may inhibit cellular proliferation and promote partial cell cycle arrest in cancer cell lines through interactions with the adenosine monophosphate‐activated protein kinase (AMPK) metabolism by inhibition of the AMPK/liver kinase B‐1 dependent growth pathway or reducing systemic circulating insulin levels.^42^, ^43^ We also found that the risk reduction with metformin monotherapy was site‐specific suggesting pleiotropic effects against cancer, or differences in carcinogenesis at different sites. The more significant GLP‐1 RA‐induced weight loss and improvements in glycaemic control may indirectly reduce the risk of obesity and T2D‐related cancers more potently. Moreover, in vitro studies have found that activation of the GLP‐1 receptor inhibits cell proliferation, independent of insulin and augments apoptosis.^44^ Metformin increases the plasma levels of GLP‐1 after an oral glucose load in patients with obesity, but without diabetes, suggesting that metformin could increase circulating GLP‐1 levels.^45^ Therefore, metformin may potentiate the glucose‐lowering and weight loss benefits of GLP‐1 RAs and may explain the synergistic effects of both drugs in relation to reduced cancer incidence in our study.

We are currently witnessing dual epidemics of obesity and T2D, and with the higher cancer incidence observed in these groups, an increased overall cancer incidence in the general population.^46^ One study reported that 5.7% of all incident cancers in 2012 were related to T2D and obesity, with 25.8% of diabetes‐related cancers, and 31.9% of adiposity‐related cancers being attributed to these two factors.^47^ An even more alarming public health trend globally is the younger onset of obesity, T2D (<40 years of age), and cancer incidence, particularly adiposity‐related cancer.^48^ Cancer incidence rates have increased in all age groups; however, the greatest increase is in people <50 years old. By way of illustration, colorectal cancer with a previous peak incidence of 67 years now has an increasing prevalence in young adults with more cases being identified in those <50 years according to data from the National Cancer Institute. Our findings have implications with the results of the stratified analyses highlighting that the cancer‐preventative effects of metformin and GLP‐1RAs were most potent in this target (younger) population.

Metformin is usually the first‐line drug for T2D, with GLP‐1 RAs prescribed later in the management pathway. However, there remains a significant proportion of eligible patients not prescribed either drug; according to a US study reporting only 5%–6% of eligible patients were prescribed GLP‐1 RAs.^49^ The reasons for this omission are multi‐factorial but may relate to their clinical guideline position, their injectable route of administration, higher acquisition cost, associated gastrointestinal side effects, and the concern regarding the risk of pancreatitis. Only three‐quarters of general practitioners believed that they had sufficient knowledge to prescribe GLP‐1 RAs suggesting barriers of knowledge remain a limiting factor.^50^ This additional cancer‐preventative benefit for dual therapy including GLP‐RA therapy in patients with T2D, living with overweight/obesity, provides a further rationale for their more widespread adoption. Greater clinician education and more widespread multi‐disciplinary working with primary care providers, endocrinologists, weight loss services, and specialist nurse practitioners can enhance this therapeutic gap.

We acknowledge limitations to this study. Firstly, comparisons are not randomised or controlled with real‐world data; therefore we are unable to prove causation. Secondly, as data are extracted from pooled electronic health records from an administrative database, there may be missing data. As with any large database study, there may be residual bias confounding due to limitations in coding for potential confounding variables. Unmeasured confounders, including family history, and cancer screening intensity, may bias results. Surveillance and detection bias may also differ across treatment groups. Because competing‐risk models are not available within the TriNetX platform, we are unable to account for death as a competing event; thus, cancer incidence may be overestimated in the presence of lower all‐cause mortality. Incidence rates per 1000 person‐years are reported alongside hazard ratios to enhance interpretation. Moreover, BMI poorly reflects body composition, overestimating adiposity in people with high skeletal muscle mass, and underestimating adiposity in the elderly, white, and younger populations.^51^ In addition, the relatively short follow‐up time of 5 years may be insufficient to detect the full chemoprotective effects of metformin and GLP‐1 RAs on cancer incidence. The relative novelty of GLP‐1 RAs restricted this follow‐up time. Further, although patients were matched for BMI and HbA1c, we were unable to accurately assess changes in these variables from baseline. We attempted to reduce the impact of unknown confounding through calculation of E‐values as a quantitative bias analysis to assist readers in the interpretation of the strength of our result. Finally, we were not able to discern whether patients were prescribed/treated with other glucose‐lowering medications during the follow‐up period, nor could we assess accurately whether patients changed agents within the GLP‐1 RA drug class.

In conclusion, in patients with T2D, individual use of metformin and GLP‐1 RAs, and especially combination therapy, was associated with a lower incidence of all adiposity‐related cancers and mortality. Considering the worldwide pandemics of obesity, T2D and these higher rates of cancer incidence, particularly in younger people, explorations of effective cancer prevention strategies are of great clinical and public health importance.

## CONFLICT OF INTEREST STATEMENT

Matthew Anson receives a fellowship from the Novo Nordisk UK research foundation and JDRF. CAR has acted as a consultant for Boehringer Ingelheim, and has received grant funding from Unilever. Daniel J Cuthbertson has received investigator‐initiated grants from Astra Zeneca and Novo Nordisk, support for education from Perspectum with any financial remuneration from pharmaceutical company consultation made to the University of Liverpool. GHI is an employee of TriNetX LLC. Uazman Alam has received honoraria from Procter & Gamble, Viatris, Grunenthal and Sanofi for educational meetings and funding for attendance to an educational meeting from Diiachi Sankyo. Uazman Alam has also received investigator‐led funding by Procter & Gamble and is a council member of the Royal Society of Medicine's Vascular, Lipid & Metabolic Medicine Section. All other authors declare that there are no financial relationships or activities that might bias, or be perceived to bias, their contribution to this manuscript.

## Supporting information


**TABLE S1.** Definitions for all baseline diagnoses, covariates, and outcomes.
**TABLE S2.** Outcomes for specific obesity‐related cancers with metformin vs. DPP4i treatment. Where the total number of events for the respective cancer was below 10, for patient confidentiality reasons, TriNetX displays the number as 10.
**TABLE S3.** Outcomes for specific obesity‐related cancers with GLP‐1 receptor agonist vs. DPP4i treatment. Where the total number of events for the respective cancer was below 10, for patient confidentiality reasons, TriNetX displays the number as 10.
**TABLE S4.** Outcomes for specific obesity‐related cancers with dual metformin and GLP‐1 receptor agonist treatment. Where the total number of events for the respective cancer was below 10, for patient confidentiality reasons, TriNetX displays the number as 10.
**TABLE S5.** Stratified analyses for the outcomes of all obesity‐related cancers.
**FIGURE S1.** Forest plot stratified by body mass index.
**FIGURE S2.** Forest plot stratified by age.
**FIGURE S3.** Forest plot stratified by ethnicity.
**FIGURE S4.** Forest plot stratified by sex.
**FIGURE S5.** Forest plot stratified by alcohol excess.
**FIGURE S6.** Forest plot stratified by duration of type 2 diabetes diagnosis.

## Data Availability

The data used in this study were obtained through the TriNetX Research Network, a federated real‐world data network aggregating de‐identified electronic health record (EHR) and related clinical data from participating healthcare organisations.Each contributing site certifies that it has the necessary institutional permissions and rights to share de‐identified patient data under applicable privacy laws and institutional agreements. TriNetX applies de‐identification in accordance with the HIPAA “safe harbor” and/or “expert determination” standards (as per 164.514(a)) to ensure that no personally identifying information is retained in the dataset. The dataset is restricted to patients meeting our pre‐specified inclusion and exclusion criteria. Only those cases for which the required data elements (demographics, diagnoses, medications, laboratory values, procedures, and outcomes) were available in the TriNetX network during the study period were included.Because the data are de‐identified and delivered via a secure platform under strict access controls, no further individual patient consent was required. Access to the data is limited to authorised study personnel, and all data handling complies with institutional data security and governance policies.
