# Supplementary material for: Synergistic associations of metformin and GLP‐1 receptor agonist use with adiposity‐related cancer incidence in people living with type 2 diabetes
Source: Diabetes Obes Metab. 2025 Nov 3;28(2):946–59. doi: 10.1111/dom.70267 (PMC12803636; doi:10.1111/dom.70267)
Supplement: Supplementary file 1 — TABLE S1. Definitions for all baseline diagnoses, covariates, and outcomes. TABLE S2. Outcomes for specific obesity‐related cancers with metformin vs. DPP4i treatment. Where the total number of events for the respective cancer was below 10, for patient confidentiality reasons, TriNetX displays the number as 10. TABLE S3. Outcomes for specific obesity‐related cancers with GLP‐1 receptor agonist vs. DPP4i treatment. Where the total number of events for the respective cancer was below 10, for patient confidentiality reasons, TriNetX displays the number as 10. TABLE S4. Outcomes for specific obesity‐related cancers with dual metformin and GLP‐1 receptor agonist treatment. Where the total number of events for the respective cancer was below 10, for patient confidentiality reasons, TriNetX displays the number as 10. TABLE S5. Stratified analyses for the outcomes of all obesity‐related cancers. FIGURE S1. Forest plot stratified by body mass index. FIGURE S2. Forest plot stratified by age. FIGURE S3. Forest plot stratified by ethnicity. FIGURE S4. Forest plot stratified by sex. FIGURE S5. Forest plot stratified by alcohol excess. FIGURE S6. Forest plot stratified by duration of type 2 diabetes diagnosis. [file DOM-28-946-s001.docx]

**Supplementary Material**

| **Diagnosis** | **ICD-10 code** |
| --- | --- |
| **Inclusion and exclusion criteria** | |
| Type 2 diabetes | E11 |
| Type 1 diabetes | E10 |
| **Covariates** | |
| Ischaemic heart disease | I20-I25 |
| Cerebrovascular disease | I60-I69 |
| Peripheral vascular disease | I73 |
| Hypertension | I10 |
| Dyslipidaemia | E78 |
| Nicotine dependence | F17.2 |
| Socioeconomic hazards | Z55-Z65 |
| Neoplasm | C00-D49 |
| **Outcomes, cancers** | |
| Oral cavity | C06 |
| Oesophageal | C15 |
| Stomach | C16 |
| Colon | C18 |
| Rectal | C19 |
| Hepatocellular | C22 |
| Gallbladder | C23 |
| Pancreas | C25 |
| Sinus | C31 |
| Connective tissue | C49 |
| Breast | C50 |
| Vulva | C51 |
| Cervix | C53 |
| Uterus | C55 |
| Ovary | C56 |
| Penis | C60 |
| Kidney | C64 |
| Brain | C71 |
| Thyroid | C73 |
| Adrenal | C74 |
| Parathyroid | C75.0 |
| Pituitary | C75.1 |
| Head and neck | C76.0 |
| Multiple myeloma | C90 |
| Melanoma | D03 |
| **Sensitivity analysis** | |
| Alcohol related disorders | F10 |

**Supplementary Table 1** Definitions for all baseline diagnoses, covariates, and outcomes

|  | **Sample size** | **Outcome (n)** | **5-year survival probability (%)** | **Hazard ratio (95% confidence interval)** | **Log-Rank test** | **P value** | **E-value** |
| --- | --- | --- | --- | --- | --- | --- | --- |
| **Metformin *vs.* DPP4i** | | | | | | | |
| **Gastrointestinal carcinomas** | | | | | | | |
| ***Hepatocellular*** | | | | | | | |
| Reference | 88,786 | 483 | 99.3 | 1.00 (1.00, 1.00) | | | |
| Metformin | 88,786 | 384 | 99.4 | **0.79 (0.69, 0.90)** | 12.4 | <0.01 | 1.85 |
| ***Colorectal*** | | | | | | | |
| Reference | 88,786 | 667 | 99.0 | 1.00 (1.00, 1.00) | | | |
| Metformin | 88,786 | 666 | 99.0 | 0.99 (0.89, 1.10) | 0.1 | 0.83 | 1.00 |
| ***Gallbladder*** | | | | | | | |
| Reference | 88,786 | 38 | 99.9 | 1.00 (1.00, 1.00) | | | |
| Metformin | 88,786 | 33 | >99.9 | 0.86 (0.54, 1.38) | 0.4 | 0.54 | 1.00 |
| ***Pancreatic*** | | | | | | | |
| Reference | 88,786 | 329 | 99.5 | 1.00 (1.00, 1.00) | | | |
| Metformin | 88,786 | 403 | 99.4 | **1.22 (1.05, 1.41)** | 7.1 | 0.01 | 1.74 |
| ***Oesophageal*** | | | | | | | |
| Reference | 88,786 | 120 | 99.8 | 1.00 (1.00, 1.00) | | | |
| Metformin | 88,786 | 116 | 99.8 | 0.96 (0.74, 1.23) | 2.3 | 0.78 | 1.00 |
| ***Gastric*** | | | | | | | |
| Reference | 88,786 | 141 | 99.8 | 1.00 (1.00, 1.00) | | | |
| Metformin | 88,786 | 130 | 99.8 | 0.91 (0.72, 1.16) | 0.6 | 0.46 | 1.00 |
| **Genitourinary and female reproductive health carcinomas** | | | | | | | |
| ***Vulval*** | | | | | | | |
| Reference | 88,786 | 25 | >99.9 | 1.00 (1.00, 1.00) | | | |
| Metformin | 88,786 | 34 | 99.9 | 1.34 (0.80, 2.24) | 1.2 | 0.27 | 1.00 |
| ***Cervical*** | | | | | | | |
| Reference | 88,786 | 66 | 99.9 | 1.00 (1.00, 1.00) | | | |
| Metformin | 88,786 | 77 | 99.9 | 1.15 (0.83, 1.60) | 0.7 | 0.40 | 1.00 |
| ***Uterine*** | | | | | | | |
| Reference | 88,786 | 266 | 99.6 | 1.00 (1.00, 1.00) | | | |
| Metformin | 88,786 | 291 | 99.5 | 1.08 (0.92, 1.28) | 0.88 | 0.35 | 1.00 |
| ***Breast*** | | | | | | | |
| Reference | 88,786 | 907 | 98.6 | 1.00 (1.00, 1.00) | | | |
| Metformin | 88,786 | 1028 | 98.4 | **1.12 (1.03, 1.23)** | 6.4 | 0.01 | 1.49 |
| ***Ovarian*** | | | | | | | |
| Reference | 88,786 | 102 | 99.8 | 1.00 (1.00, 1.00) | | | |
| Metformin | 88,786 | 143 | 99.8 | **1.39 (1.08, 1.79)** | 6.5 | 0.01 | 2.13 |
| ***Penile*** | | | | | | | |
| Reference | 88,786 | 15 | <99.9 | 1.00 (1.00, 1.00) | | | |
| Metformin | 88,786 | 19 | <99.9 | 1.26 (0.64, 2.47) | 0.4 | 0.51 | 1.00 |
| ***Renal*** | | | | | | | |
| Reference | 88,786 | 652 | 99.0 | 1.00 (1.00, 1.00) | | | |
| Metformin | 88,786 | 404 | 99.4 | **0.61 (0.54, 0.70)** | 60.7 | <0.01 | 2.66 |
| **Endocrine gland carcinomas** | | | | | | | |
| ***Thyroid*** | | | | | | | |
| Reference | 88,786 | 204 | 99.7 | 1.00 (1.00, 1.00) | | | |
| Metformin | 88,786 | 204 | 99.7 | 0.99 (0.82, 1.21) | <0.1 | 0.96 | 1.00 |
| ***Parathyroid*** | | | | | | | |
| Reference | 88,786 | 10 | >99.9 | 1.00 (1.00, 1.00) | | | |
| Metformin | 88,786 | 10 | >99.9 | 0.33 (0.03, 3.17) | 1.0 | 0.31 | 1.00 |
| ***Adrenal*** | | | | | | | |
| Reference | 88,786 | 22 | >99.9 | 1.00 (1.00, 1.00) | | | |
| Metformin | 88,786 | 21 | >99.9 | 0.95 (0.52, 1.72) | <0.01 | 0.86 | 1.00 |
| ***Pituitary*** | | | | | | | |
| Reference | 88,786 | 10 | >99.9 | 1.00 (1.00, 1.00) | | | |
| Metformin | 88,786 | 10 | >99.9 | 1.33 (0.30, 5.94) | 0.1 | 0.71 | 1.00 |
| **Other carcinomas** | | | | | | | |
| ***Oral cavity*** | | | | | | | |
| Reference | 88,786 | 52 | 99.9 | 1.00 (1.00, 1.00) | | | |
| Metformin | 88,786 | 51 | 99.9 | 0.97 (0.66, 1.43) | 0.02 | 0.88 | 1.00 |
| ***Head and neck*** | | | | | | | |
| Reference | 88,786 | 153 | 99.8 | 1.00 (1.00, 1.00) | | | |
| Metformin | 88,786 | 149 | 99.8 | 0.97 (0.77, 1.21) | 0.1 | 0.77 | 1.00 |
| ***Sinus*** | | | | | | | |
| Reference | 88,786 | 18 | >99.9 | 1.00 (1.00, 1.00) | | | |
| Metformin | 88,786 | 10 | >99.9 | 0.50 (0.22, 1.10) | 3.1 | 0.08 | 1.00 |
| ***Connective tissue*** | | | | | | | |
| Reference | 88,786 | 164 | 99.8 | 1.00 (1.00, 1.00) | | | |
| Metformin | 88,786 | 189 | 99.7 | 1.14 (0.93, 1.41) | 1.6 | 0.21 | 1.00 |
| ***Brain*** | | | | | | | |
| Reference | 88,786 | 112 | 99.8 | 1.00 (1.00, 1.00) | | | |
| Metformin | 88,786 | 130 | 99.8 | 1.15 (0.90, 1.48) | 1.21 | 0.27 | 1.00 |
| ***Bone marrow*** | | | | | | | |
| Reference | 88,786 | 299 | 99.5 | 1.00 (1.00, 1.00) | | | |
| Metformin | 88,786 | 211 | 99.7 | **0.70 (0.59, 0.83)** | 16.0 | <0.01 | 2.21 |
| ***Melanoma*** | | | | | | | |
| Reference | 88,786 | 192 | 99.7 | 1.00 (1.00, 1.00) | | | |
| Metformin | 88,786 | 185 | 99.7 | 0.95 (0.78, 1.17) | 0.2 | 0.65 | 1.00 |

**Supplementary Table 2** Outcomes for specific obesity-related cancers with metformin *vs.* DPP4i treatment. Where the total number of events for the respective cancer was below 10, for patient confidentiality reasons, TriNetX displays the number as 10.

|  | **Sample size** | **Outcome (n)** | **5-year survival probability (%)** | **Hazard ratio (95% confidence interval)** | **Log-Rank test** | **P value** | **E-value** |
| --- | --- | --- | --- | --- | --- | --- | --- |
| **GLP-1 RA *vs.* DPP4i** | | | | | | | |
| **Gastrointestinal carcinomas** | | | | | | | |
| ***Hepatocellular*** | | | | | | | |
| Reference | 112,735 | 489 | 99.4 | 1.00 (1.00, 1.00) | | | |
| Metformin | 112,735 | 389 | 99.6 | **0.73 (0.64, 0.84)** | 20.8 | <0.01 | 2.08 |
| ***Colorectal*** | | | | | | | |
| Reference | 112,735 | 597 | 99.3 | 1.00 (1.00, 1.00) | | | |
| Metformin | 112,735 | 483 | 99.5 | **0.75 (0.66, 0.84)** | 22.5 | <0.01 | 2.00 |
| ***Gallbladder*** | | | | | | | |
| Reference | 112,735 | 32 | <99.9 | 1.00 (1.00, 1.00) | | | |
| Metformin | 112,735 | 19 | <99.9 | **0.55 (0.31, 0.97)** | 4.3 | 0.04 | 3.04 |
| ***Pancreatic*** | | | | | | | |
| Reference | 112,735 | 407 | 99.5 | 1.00 (1.00, 1.00) | | | |
| Metformin | 112,735 | 320 | 99.7 | **0.73 (0.63, 0.85)** | 17.8 | <0.01 | 2.08 |
| ***Oesophageal*** | | | | | | | |
| Reference | 112,735 | 128 | 99.9 | 1.00 (1.00, 1.00) | | | |
| Metformin | 112,735 | 98 | 99.9 | **0.71 (0.54, 0.92)** | 6.8 | 0.01 | 2.17 |
| ***Gastric*** | | | | | | | |
| Reference | 112,735 | 140 | 99.8 | 1.00 (1.00, 1.00) | | | |
| Metformin | 112,735 | 114 | 99.9 | **0.75 (0.59, 0.96)** | 5.2 | 0.02 | 2.00 |
| **Genitourinary and female reproductive health carcinomas** | | | | | | | |
| ***Vulval*** | | | | | | | |
| Reference | 112,735 | 25 | <99.9 | 1.00 (1.00, 1.00) | | | |
| Metformin | 112,735 | 40 | <99.9 | 1.47 (0.89, 2.43) | 2.3 | 0.13 | 1.00 |
| ***Cervical*** | | | | | | | |
| Reference | 112,735 | 74 | 99.9 | 1.00 (1.00, 1.00) | | | |
| Metformin | 112,735 | 98 | 99.9 | 1.23 (0.91, 1.67) | 1.84 | 0.18 | 1.00 |
| ***Uterine*** | | | | | | | |
| Reference | 112,735 | 382 | 99.6 | 1.00 (1.00, 1.00) |  |  |  |
| Metformin | 112,735 | 421 | 99.6 | 1.02 (0.89, 1.17) | 0.1 | 0.78 | 1.00 |
| ***Breast*** | | | | | | | |
| Reference | 112,735 | 1067 | 98.8 | 1.00 (1.00, 1.00) | | | |
| Metformin | 112,735 | 1169 | 98.8 | 1.01 (0.93, 1.10) | 0.1 | 0.75 | 1.00 |
| ***Ovarian*** | | | | | | | |
| Reference | 112,735 | 161 | 99.8 | 1.00 (1.00, 1.00) | | | |
| Metformin | 112,735 | 148 | 99.8 | 0.85 (0.68, 1.06) | 2.1 | 0.15 | 1.00 |
| ***Penile*** | | | | | | | |
| Reference | 112,735 | 17 | <99.9 | 1.00 (1.00, 1.00) | | | |
| Metformin | 112,735 | 10 | <99.9 | 0.55 (0.25, 1.20) | 2.4 | 0.12 | 1.00 |
| ***Renal*** | | | | | | | |
| Reference | 112,735 | 561 | 99.4 | 1.00 (1.00, 1.00) | | | |
| Metformin | 112,735 | 520 | 99.5 | **0.86 (0.76, 0.97)** | 6.1 | 0.01 | 1.60 |
| **Endocrine gland carcinomas** | | | | | | | |
| ***Thyroid*** | | | | | | | |
| Reference | 112,735 | 307 | 99.7 | 1.00 (1.00, 1.00) | | | |
| Metformin | 112,735 | 330 | 99.7 | 1.00 (0.86, 1.17) | <0.1 | 0.98 | 1.00 |
| ***Parathyroid*** | | | | | | | |
| Reference | 112,735 | 10 | >99.9 | 1.00 (1.00, 1.00) | | | |
| Metformin | 112,735 | 10 | >99.9 | 1.15 (0.31, 4.28) | <0.1 | 0.84 | 1.00 |
| ***Adrenal*** | | | | | | | |
| Reference | 112,735 | 31 | >99.9 | 1.00 (1.00, 1.00) | | | |
| Metformin | 112,735 | 24 | >99.9 | 0.72 (0.42, 1.22) | 1.5 | 0.22 | 1.00 |
| ***Pituitary*** | | | | | | | |
| Reference | 112,735 | 10 | >99.9 | 1.00 (1.00, 1.00) | | | |
| Metformin | 112,735 | 10 | >99.9 | 1.30 (0.50, 3.43) | 0.3 | 0.59 | 1.00 |
| **Other carcinomas** | | | | | | | |
| ***Oral cavity*** | | | | | | | |
| Reference | 112,735 | 69 | 99.9 | 1.00 (1.00, 1.00) | | | |
| Metformin | 112,735 | 48 | 99.9 | **0.65 (0.45, 0.93)** | 5.5 | 0.02 | 2.45 |
| ***Head and neck*** | | | | | | | |
| Reference | 112,735 | 158 | 99.8 | 1.00 (1.00, 1.00) | | | |
| Metformin | 112,735 | 115 | 99.9 | **0.68 (0.53, 0.86)** | 10.3 | <0.01 | 2.30 |
| ***Sinus*** | | | | | | | |
| Reference | 112,735 | 14 | >99.9 | 1.00 (1.00, 1.00) | | | |
| Metformin | 112,735 | 13 | >99.9 | 0.87 (0.41, 1.85) | 0.1 | 0.71 | 1.00 |
| ***Connective tissue*** | | | | | | | |
| Reference | 112,735 | 179 | 99.8 | 1.00 (1.00, 1.00) | | | |
| Metformin | 112,735 | 184 | 99.8 | 0.95 (0.78, 1.17) | 0.2 | 0.64 | 1.00 |
| ***Brain*** | | | | | | | |
| Reference | 112,735 | 163 | 99.8 | 1.00 (1.00, 1.00) | | | |
| Metformin | 112,735 | 108 | 99.9 | **0.62 (0.49, 0.79)** | 15.2 | <0.01 | 2.61 |
| ***Bone marrow*** | | | | | | | |
| Reference | 112,735 | 214 | 99.8 | 1.00 (1.00, 1.00) | | | |
| Metformin | 112,735 | 197 | 99.8 | 0.85 (0.70, 1.03) | 2.8 | 0.09 | 1.00 |
| ***Melanoma*** | | | | | | | |
| Reference | 112,735 | 189 | 99.8 | 1.00 (1.00, 1.00) | | | |
| Metformin | 112,735 | 178 | 99.8 | 0.87 (0.71, 1.07) | 1.7 | 0.19 | 1.00 |

**Supplementary Table 3** Outcomes for specific obesity-related cancers with GLP-1 receptor agonist *vs.* DPP4i treatment. Where the total number of events for the respective cancer was below 10, for patient confidentiality reasons, TriNetX displays the number as 10.

|  | **Sample size** | **Outcome (n)** | **5-year survival probability (%)** | **Hazard ratio (95% confidence interval)** | **Log-Rank test** | **P value** | **E-value** |
| --- | --- | --- | --- | --- | --- | --- | --- |
| **Dual metformin and GLP-1 RA treatment *vs.* DPP4i** | | | | | | | |
| **Gastrointestinal carcinomas** | | | | | | | |
| ***Hepatocellular*** | | | | | | | |
| Reference | 36,347 | 216 | 99.2 | 1.00 (1.00, 1.00) | | | |
| Metformin | 36,347 | 98 | 99.7 | **0.38 (0.30, 0.48)** | 69.8 | <0.01 | 2.63 |
| ***Colorectal*** | | | | | | | |
| Reference | 36,347 | 228 | 99.2 | 1.00 (1.00, 1.00) | | | |
| Metformin | 36,347 | 148 | 99.5 | **0.54 (0.44, 0.66)** | 35.9 | <0.01 | 3.11 |
| ***Gallbladder*** | | | | | | | |
| Reference | 36,347 | 13 | >99.9 | 1.00 (1.00, 1.00) | | | |
| Metformin | 36,347 | 10 | >99.9 | 0.39 (0.15, 1.03) | 3.84 | 0.05 | 1.00 |
| ***Pancreatic*** | | | | | | | |
| Reference | 36,347 | 119 | 99.6 | 1.00 (1.00, 1.00) | | | |
| Metformin | 36,347 | 98 | 99.7 | **0.69 (0.53, 0.91)** | 7.2 | 0.01 | 2.26 |
| ***Oesophageal*** | | | | | | | |
| Reference | 36,347 | 47 | 99.8 | 1.00 (1.00, 1.00) | | | |
| Metformin | 36,347 | 21 | 99.9 | **0.37 (0.22, 0.63)** | 15.2 | <0.01 | 4.85 |
| ***Gastric*** | | | | | | | |
| Reference | 36,347 | 43 | 99.8 | 1.00 (1.00, 1.00) | | | |
| Metformin | 36,347 | 29 | 99.9 | **0.56 (0.35, 0.90)** | 5.8 | 0.02 | 2.97 |
| **Genitourinary and female reproductive health carcinomas** | | | | | | | |
| ***Vulval*** | | | | | | | |
| Reference | 36,347 | 10 | >99.9 | 1.00 (1.00, 1.00) | | | |
| Metformin | 36,347 | 12 | >99.9 | 1.07 (0.45, 2.54) | <0.1 | 0.88 | 1.00 |
| ***Cervical*** | | | | | | | |
| Reference | 36,347 | 29 | 99.9 | 1.00 (1.00, 1.00) | | | |
| Metformin | 36,347 | 23 | 99.9 | 0.66 (0.38, 1.14) | 2.3 | 0.13 | 1.00 |
| ***Uterine*** | | | | | | | |
| Reference | 36,347 | 138 | 99.5 | 1.00 (1.00, 1.00) | | | |
| Metformin | 36,347 | 95 | 99.7 | **0.57 (0.44, 0.74)** | 18.5 | <0.01 | 2.90 |
| ***Breast*** | | | | | | | |
| Reference | 36,347 | 362 | 99.6 | 1.00 (1.00, 1.00) | | | |
| Metformin | 36,347 | 354 | 99.8 | **0.84 (0.73, 0.97)** | 5.5 | 0.02 | 1.67 |
| ***Ovarian*** | | | | | | | |
| Reference | 36,347 | 45 | 99.8 | 1.00 (1.00, 1.00) | | | |
| Metformin | 36,347 | 48 | 99.8 | 0.88 (0.58, 1.32) | 0.4 | 0.53 | 1.00 |
| ***Penile*** | | | | | | | |
| Reference | 36,347 | 10 | <99.9 | 1.00 (1.00, 1.00) | | | |
| Metformin | 36,347 | 10 | <99.9 | 0.85 (0.27, 2.63) | 0.1 | 0.78 | 1.00 |
| ***Renal*** | | | | | | | |
| Reference | 36,347 | 280 | 99.0 | 1.00 (1.00, 1.00) | | | |
| Metformin | 36,347 | 164 | 99.5 | **0.49 (0.40, 0.59)** | 56.4 | <0.01 | 3.50 |
| **Endocrine gland carcinomas** | | | | | | | |
| ***Thyroid*** | | | | | | | |
| Reference | 36,347 | 90 | 99.7 | 1.00 (1.00, 1.00) | | | |
| Metformin | 36,347 | 109 | 99.7 | 1.01 (0.77, 1.35) | <0.1 | 0.90 | 1.00 |
| ***Parathyroid*** | | | | | | | |
| Reference | 36,347 | 10 | >99.9 | 1.00 (1.00, 1.00) | | | |
| Metformin | 36,347 | 10 | >99.9 | 1.56 (0.14, 17.19) | 0.1 | 0.72 | 1.00 |
| ***Adrenal*** | | | | | | | |
| Reference | 36,347 | 13 | >99.9 | 1.00 (1.00, 1.00) | | | |
| Metformin | 36,347 | 10 | >99.9 | 0.39 (0.15, 1.02) | 4.0 | 0.05 | 1.00 |
| ***Pituitary*** | | | | | | | |
| Reference | 36,347 | 10 | >99.9 | 1.00 (1.00, 1.00) | | | |
| Metformin | 36,347 | 10 | >99.9 | 0.81 (0.11, 5.75) | <0.1 | 0.83 | 1.00 |
| **Other carcinomas** | | | | | | | |
| ***Oral cavity*** | | | | | | | |
| Reference | 36,347 | 16 | 99.9 | 1.00 (1.00, 1.00) | | | |
| Metformin | 36,347 | 14 | >99.9 | 0.74 (0.36, 1.53) | 0.7 | 0.42 | 1.00 |
| ***Head and neck*** | | | | | | | |
| Reference | 36,347 | 68 | 99.7 | 1.00 (1.00, 1.00) | | | |
| Metformin | 36,347 | 44 | 99.9 | **0.54 (0.37, 0.79)** | 10.3 | <0.01 | 3.11 |
| ***Sinus*** | | | | | | | |
| Reference | 36,347 | 10 | >99.9 | 1.00 (1.00, 1.00) | | | |
| Metformin | 36,347 | 10 | >99.9 | 0.39 (0.10, 1.51) | 2.0 | 0.16 | 1.00 |
| ***Connective tissue*** | | | | | | | |
| Reference | 36,347 | 71 | 99.7 | 1.00 (1.00, 1.00) | | | |
| Metformin | 36,347 | 62 | 99.8 | 0.74 (0.52, 1.04) | 3.1 | 0.08 | 1.00 |
| ***Brain*** | | | | | | | |
| Reference | 36,347 | 55 | 99.8 | 1.00 (1.00, 1.00) | | | |
| Metformin | 36,347 | 32 | 99.9 | **0.50 (0.32, 0.77)** | 10.3 | <0.01 | 3.41 |
| ***Bone marrow*** | | | | | | | |
| Reference | 36,347 | 93 | 99.7 | 1.00 (1.00, 1.00) | | | |
| Metformin | 36,347 | 58 | 99.8 | **0.51 (0.37, 0.71)** | 16.7 | <0.01 | 3.33 |
| ***Melanoma*** | | | | | | | |
| Reference | 36,347 | 70 | 99.7 | 1.00 (1.00, 1.00) | | | |
| Metformin | 36,347 | 50 | 99.8 | **0.59 (0.41, 0.85)** | 8.1 | <0.01 | 2.78 |

**Supplementary Table 4** Outcomes for specific obesity-related cancers with dual metformin and GLP-1 receptor agonist treatment. Where the total number of events for the respective cancer was below 10, for patient confidentiality reasons, TriNetX displays the number as 10.

| **Stratification** | **Hazard ratio [95% confidence interval]** |
| --- | --- |
| **Metformin vs DPP4i** | |
| ***Body mass index*** | |
| Obesity, BMI >30kg/m^2^ | 0.94 [0.87, 1.01] |
| No obesity, BMI <30kg/m^2^ | 0.99 [0.91, 1.08] |
| ***Age*** | |
| Older adults, >60 years | **0.96 [0.92, 0.99]** |
| Younger adults, <60 years | **0.71 [0.59, 0.85]** |
| ***Ethnicity*** | |
| White | 0.96 [0.91, 1.01] |
| Non-white | 0.94 [0.88, 1.01] |
| ***Sex*** | |
| Male | **0.93 [0.87, 0.99]** |
| Female | 1.00 [0.94, 1.06] |
| ***Alcohol*** | |
| Alcohol excess excluded | **0.96 [0.92, 0.99]** |
| ***Diabetes duration*** | |
| Diabetes diagnosis >10 years ago | 0.95 [0.88, 1.01] |
| Diabetes diagnosis <10 years ago | **0.94 [0.90. 0.98]** |
| ***Drug adherence*** | |
| Drug adherence for >1 year | **0.87 [0.82, 0.92]** |
| ***Pre-index event exclusion*** | |
| Exclusion of patients with cancer occurring within 6 months of the index event | **0.94 [0.90, 0.97]** |
| **GLP-1 RA vs DPP4i** | |
| ***Body mass index*** | |
| Obesity, BMI >30kg/m^2^ | **0.87 [0.81, 0.92]** |
| No obesity, BMI <30kg/m^2^ | **0.86 [0.75, 0.98]** |
| ***Age*** | |
| Older adults, >60 years | **0.85 [0.81, 0.89]** |
| Younger adults, <60 years | **0.77 [0.69, 0.86]** |
| ***Ethnicity*** | |
| White | **0.83 [0.80, 0.88]** |
| Non-white | **0.87 [0.81, 0.94]** |
| ***Sex*** | |
| Male | **0.71 [0.67,0.76]** |
| Female | **0.88 [0.84, 0.93]** |
| ***Specific drug*** | |
| Semaglutide | **0.78 [0.65, 0.94]** |
| Liraglutide | **0.88 [0.83, 0.94]** |
| ***Alcohol*** | |
| Alcohol excess excluded | **0.85 [0.81, 0.89]** |
| ***Diabetes duration*** | |
| Diabetes diagnosis >10 years ago | **0.79 [0.74, 0.85]** |
| Diabetes diagnosis <10 years ago | **0.84 [0.80, 0.88]** |
| ***Drug adherence*** | |
| Drug adherence for >1 year | **0.73 [0.67, 0.79]** |
| ***Pre-index event exclusion*** | |
| Exclusion of patients with cancer occurring within 6 months of the index event | **0.85 [0.82, 0.88]** |
| **Dual metformin and GLP-1 RA treatment vs DPP4i** | |
| ***Body mass index*** | |
| Obesity, BMI >30kg/m^2^ | **0.67 [0.59, 0.75]** |
| No obesity, BMI <30kg/m^2^ | **0.62 [0.51, 0.75]** |
| ***Age*** | |
| Older adults, >60 years | **0.65 [0.61, 0.70]** |
| Younger adults, <60 years | **0.58 [0.46, 0.73]** |
| ***Ethnicity*** | |
| White | **0.61 [0.56, 0.67]** |
| Non-white | **0.60 [0.53, 0.69]** |
| ***Sex*** | |
| Male | **0.54 [0.48, 0.61]** |
| Female | **0.72 [0.66, 0.80]** |
| ***Alcohol*** | |
| Alcohol excess excluded | **0.63 [0.58, 0.68]** |
| ***Diabetes duration*** | |
| Diabetes diagnosis >10 years ago | **0.53 [0.46, 0.60]** |
| Diabetes diagnosis <10 years ago | **0.62 [0.58, 0.67]** |
| ***Drug adherence*** | |
| Drug adherence for >1 year | **0.52 [0.48, 0.57]** |
| ***Pre-index event exclusion*** | |
| Exclusion of patients with cancer occurring within 6 months of the index event | **0.65 [0.62, 0.70]** |

**Supplementary Table 5** Stratified analyses for the outcomes of all obesity-related cancers

**
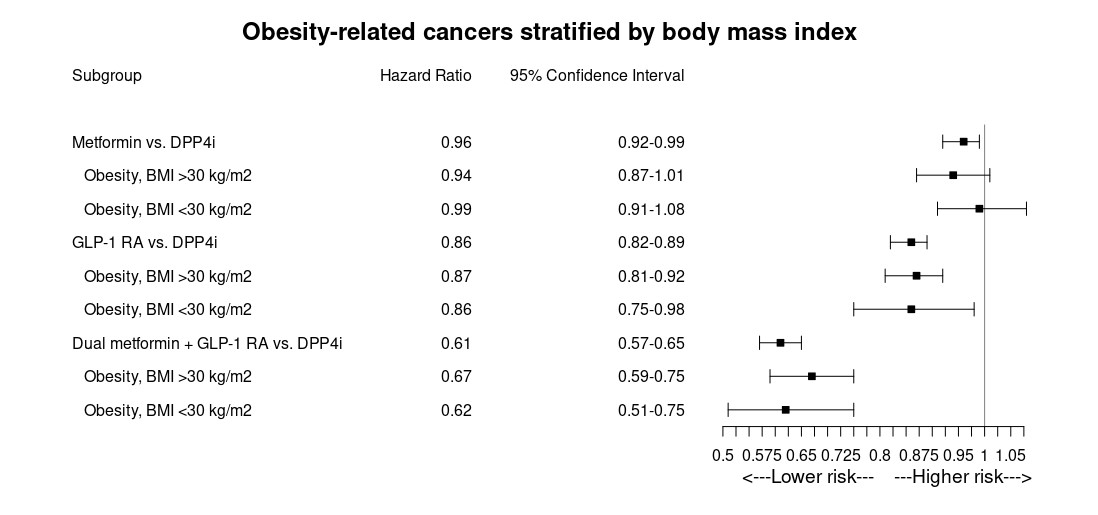
Supplementary Figure 1** Forest plot stratified by body mass index


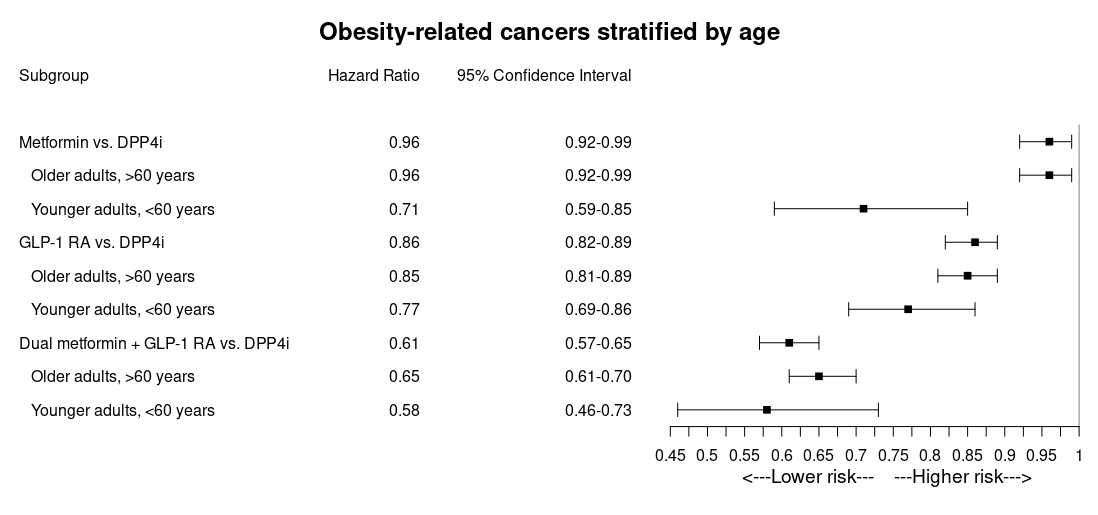


**Supplementary Figure 2** Forest plot stratified by age


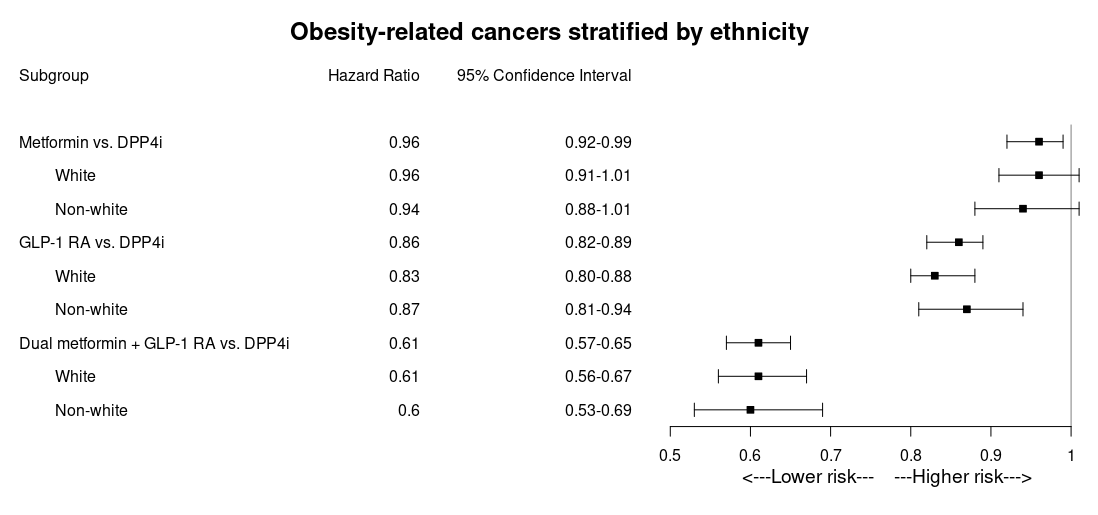
**Supplementary Figure 3** Forest plot stratified by ethnicity


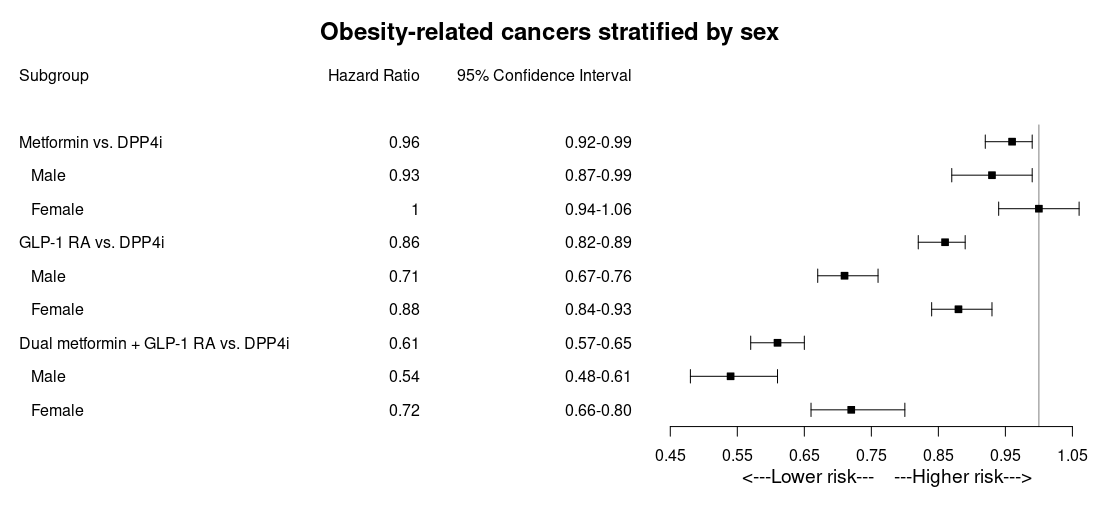


**Supplementary Figure 4** Forest plot stratified by sex


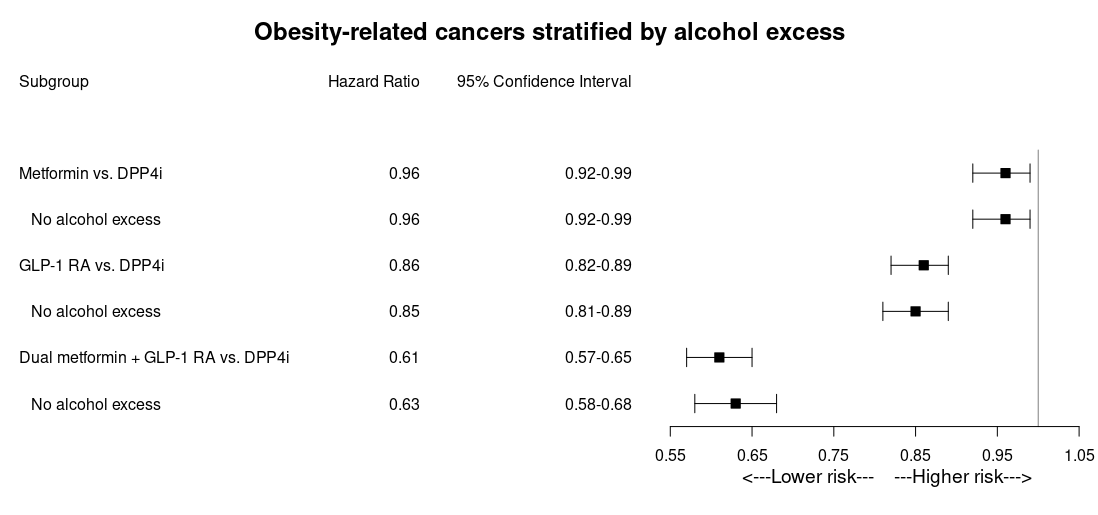


**Supplementary Figure 5** Forest plot stratified by alcohol excess


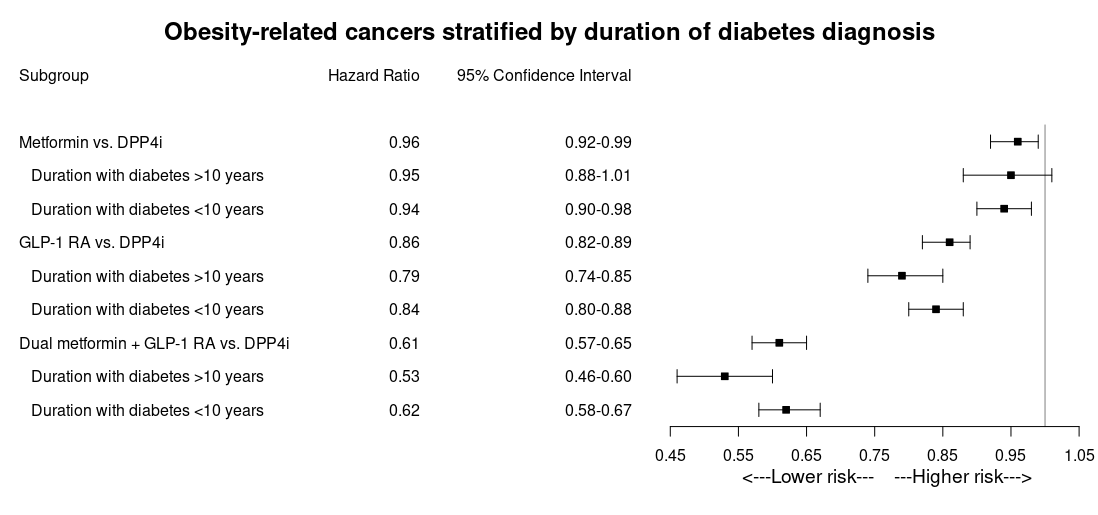


**Supplementary Figure 6** Forest plot stratified by duration of type 2 diabetes diagnosis
